# Assessment of dermal exposure to pesticides among farmers using dosimeter and hand washing methods

**DOI:** 10.3389/fpubh.2022.957774

**Published:** 2022-08-24

**Authors:** Summaiya Lari, Padmaja R. Jonnalagadda, Praveen Yamagani, Srujana Medithi, Janardhan Vanka, Arun Pandiyan, Mohan Naidu, Babban Jee

**Affiliations:** ^1^Food Safety Division, ICMR-National Institute of Nutrition, Hyderabad, Telangana, India; ^2^Department of Nutrition and Dietetics, Symbiosis Institute of Health Sciences, Symbiosis International Deemed University, Pune, India; ^3^Department of Health Research, Ministry of Health and Family Welfare, Government of India, New Delhi, India

**Keywords:** pesticides, personal protective equipment, risk assessment, potential dermal exposure, patch dosimeter, skin wiping, occupational exposure

## Abstract

Inappropriate use of pesticides followed by unsafe handling practices to control the insect infestation among the farming groups in developing countries has resulted in a high exposure risk. The use of personal protective equipment is also negligible among Indian farmers due to their affordability to access the same. Very little research has been conducted to establish an exposure assessment procedure through dermal penetration of pesticide residues. Therefore, to quantify the contamination of pesticide residues through dermal exposure along with detailed field observations and pesticide management practices, a field study was conducted in Rangareddy district, Telangana, Southern India, to assess the dermal exposure based on dosimeter and hand washing methods. The analytical method was modified and validated in-house for performance parameters such as limit of detection, quantification, linear range, recovery, and precision. The potential dermal exposure values ranged from 0.15 to 13.45 μg, while a reduction was found in exposure levels as actual dermal exposure values ranged from 0 to 0.629 μg. Contamination through hand washing was the major contributor to overall dermal exposure. Statistical analysis revealed a significant difference in the exposed dermal regions of the leg and torso after the use of PPE. Penetration factor for each anatomical region and risk evaluation in terms of the Margin of Safety implies unsafe handling of pesticides. The findings of the present study confirm the increased exposure to organophosphate pesticides among operators and highlight the importance of the use of protective measures, especially among those that focus on dermal exposure mitigation.

## Introduction

Decades ago, agrochemicals were introduced aiming at enhancing crop yields by protecting crops from pests. Due to adaptation and resistance developed by pests to chemicals and secondary pest outbreaks, every year higher amounts and new chemical compounds are used to protect crops, which not only raise the costs of food production but are also causing undesired side effects (1). This kind of non-judicious practices and unsafe use of pesticides has caused numerous problems stemming from their use through their release into the environment, while causing potential adverse effects and undesired side effects on human health (2–4). Furthermore, occupational, accidental, or intentional exposure to pesticides sometimes also resulted in hospitalization and deaths (5). Therefore, the role of exposure and the resulting risk assessment has become extremely significant, particularly for occupationally exposed groups.

Exposure to pesticides among farmers during their various preparation steps in the field applications may occur in several ways such as ingestion, inhalation, ocular, or skin contact. It is well-established that out of different routes of exposure, skin absorption is the major and relevant route of pesticide entry into the human body (6, 7). Further, as claimed by many studies, dermal exposure seemed to comprise the bulk of cumulative exposure; consequently, the protection afforded by garments or personal protective clothing must be considered essential for minimizing dermal exposure among pesticide handlers (8). However, the pesticide handlers in tropical countries including India do not usually use PPE, mainly due to their inaccessibility and discomfort associated with its use under hot and humid climatic conditions (9, 10). This in turn leads them to be more vulnerable to dermal exposure than their counterparts in temperate countries.

The developed countries, such as the European Union and North American countries, have established exposure data requirements, and do not allow a pesticide to be authorized for its use unless there is a specific data or adequate model prediction to show that, in normal use, the operator exposure levels would be below the acceptable exposure levels (11). On the contrary, in India, no such data are available, as very little research has been performed to establish exposure assessment among Indian farming groups. Moreover, most pesticide poisonings occur in developing countries because of unsafe pesticide handling practices such as poor knowledge of Good Agricultural Practices, improper training, inadequate application techniques, lack of awareness of toxicity, and negligible use of PPE (12–14).

Therefore, the importance of assessing human exposure to pesticide risk reliably has been growing. Several methods to quantify dermal exposure are available; however, they depend upon the availability of trained personnel, appropriate sophisticated equipment, elaborate chemical analyses, the inherent toxicity of pesticides, repeated exposure intensity, duration, and frequency to understand the mass of substance likely to be absorbed (15). Over the last decades, dermal exposure assessment has been the focus of research and regulations, which has resulted in the development of various methods and models addressing dermal absorption and ill health effects (16–18). However, the development of proper dermal exposure models is scarce due to different methods used to generate sound data (16).

The objectives of the present study were to evaluate the magnitude, patterns, and determinants of dermal exposure to pesticides among farmers of Rangareddy district, Telangana state, India, where the use of PPE is relatively limited and to assess the impact of the use of PPE on the minimization of exposure to pesticides. The current pesticide exposure situation in the study area selected is only representative of a large agricultural region and may generally reflect the situation in India. In the present study, field trials were conducted among six local farmers who are pesticide operators using organophosphate insecticides (OP) for the control of a variety of insect pests on different crops. An established analytical method was used for the determination of OP pesticides, and the performance parameters checked were fully validated to evaluate the dermal exposure by analyzing contamination through hand washings and the dosimeter method for patch and wipe washings. Primary objectives of the study were to identify the parameters which are likely to affect the intensity of exposure by in-field evaluation of operational modalities of the operators engaged in different farming activities through field observation and pesticide management as well as to quantify the potential dermal exposure (PDE) and actual dermal exposure (ADE) during pesticide treatment in an actual field scenario. We also aimed to evaluate the protection against pesticides by measuring skin loading rate and penetration factor (PF) and a risk indicator in terms of margin of safety (MOS).

## Materials and methods

### Study area and subjects

The study was conducted in an identified village in the Rangareddy district of Telangana state in Southern India. The annual normal rainfall of the district is 781.0 mm and the major crops grown include cotton, maize, red-gram, rice, jowar (*Sorghum*), green-gram, black-gram, castor, and other commonly grown vegetables (19). Continuous pest infestation in the region due to consecutive cultivation has led to repeated use of pesticides. From out of the larger study conducted among 217 farmers/farm workers, three subjects each of vegetables (okra, eggplant, tomato) and commercial crop (cotton) cultivators who are engaged in different farming activities were randomly selected as study operators, who also previously took part in the pesticide use survey conducted in the study area who had expressed their interest to participate.

### Ethical clearance and consent

The operators (farmers/farm workers) were made clear that the study was only in the interest of the authors' academic research to avoid any potential bias. Written consent was taken and they were also explained that they are free to decline their participation at any given point of time without any fine or penalty. The names of the participants were replaced with specific codes to use in data analyses and to ensure confidentiality. The study protocol was reviewed and approved by the ethical committee of the Indian Council of Medical Research - National Institute of Nutrition, Hyderabad, India (REF NIN Protocol number 11/I/2016).

### Field observations and pesticide management

The following information from each operator on each separate occasion was recorded using standardized field data sheets: (1) types and quantity of active ingredients handled during the day; (2) number and total duration of different phases (methods of mixing of pesticides formulation, spraying, cleaning); (3) types of work clothing (shirt/T-shirt, cotton cloth fabric, length of sleeves, trousers, shoe, scarf, if any) used; (4) use of any PPE and if not, reasons for not using; (5) crop height and farm size; (6) incidences of spills and leakages; (7) data recorded on meteorological parameters of maximum and minimum temperature (°C), relative humidity (%), wind velocity (km/h), and direction using Digital Anemometer (LM 8010, Lutron Electronic, Taiwan) two times in an hour and at every place of treatment each time on the day of samples collection; and (8) details of precautions if any followed by the operators while handling pesticides. Observations such as their re-entry into the treated fields, walking direction during spraying, incidental contaminations, and events such as breaks for equipment repairs, talking, smoking, or eating/drinking during handling of pesticides were also noted.

In the second phase of the study, the same operators were provided with a fresh set of PPE as per European Food Safety Authority guidelines and the Pesticide Handler Exposure Database (8, 20) free of cost which includes a Tychem “C” category III cover-all (DuPont™); a safety splash goggle; a cup type respirator; a pair of nitrile gloves and a pair of PVC gumboot, all procured from Usha Fire, Hyderabad (DuPont supplier, India). The operators were advised to wear the PPE provided for a period of 90 days over their regular farm clothes before handling the pesticides. The purpose of this sampling procedure is to ensure the capture of the pesticide residues that might have/not penetrated operators' clothing during farming activities and their potential absorption through their skin, followed by the adherence of residues onto their body regions which are normally not covered by their regular farm clothing.

### Monitoring of dermal exposure

A certified reference material of the pesticide—acephate, chlorpyrifos, monocrotophos, profenofos, and quinalphos and internal standard—triphenyl phosphate (TPP), were purchased from Sigma-Aldrich Chem. Pvt. Ltd., India with a certified purity of ≥97%. Pestanal grade organic solvents of acetonitrile and methanol (LC-MS grade) were purchased from Sigma-Aldrich, Merck KGaA Darmstadt, Germany with 99% purity, while formic acid (analytical grade) was purchased from Fluka Pvt. Ltd., Mumbai, India. The analytical grade reagent ethanol and anhydrous sodium chloride and sodium sulfate were purchased from Merck, Mumbai, India. The HPLC column was purchased from Agilent Technologies Pvt. Ltd., India.

The patch dosimeter, surface wipe, and hand washing methods were adopted to measure the external dermal exposure to pesticide residues among the operators (21). Trained staff has collected the samples of exposed dermal regions from the operators following the SOPs under the field conditions.

Operators were instructed to wash their hands with water before their work shift to rule out any background contamination if present. Further, the hand washing samples were collected at the end of the shift after handling the pesticides. Each operator was instructed to rinse one hand at a time approximately for at least 30 s in a Ziploc^TM^ bag made of poly-ethylene material (thickness 0.025 mm and 17.8 cm wide) containing 200 mL of ethanol (70% v/v) (22). Further, they were also provided with hypo-allergenic soap to wash their hands and water for moisturizing purposes after rinsing their hands.

Dermal exposure of other exposed body parts was also accessed by placing the patch samplers using the dosimeter method. The patch sampler was made of a surgical cotton gauze pad of approximately 1 mm thickness and 100 cm^2^ surface area, backed with an impermeable material (aluminum foil) to prevent seepage of collected residues through the patch to the skin and/or clothing. Ten of such patch samplers were attached using surgical tapes over the clothing worn by each operator (external patch) and were placed on the inner clothing under the PPE (internal patch) at different places of the exposed dermal regions. Patch samplers from corresponding exposed dermal regions were pooled and analyzed as one sample, which resulted in three patch samples per measurement (on back between shoulder blades and over the sternum [pooled as torso patch], the upper surface of right/left forearm, midway between elbow and wrist forearm [pooled as arm patch], front of right/left leg, mid-thigh and at front of right/left leg, above the ankle-below knee [pooled as leg patch]) and the same was removed using tweezers (triple-rinsed with ethanol) before changing their work clothes and after spraying tasks. This method aims to estimate the amount of a particular substance deposited on clothing/skin/penetrating through outer clothing layers.

Skin wiping technique, using surgical cotton gauze pad wetted with 2 mL of 70% ethanol as it is soluble for most compounds and causes less irritation to the skin, was employed as wipe sampler to assess the dermal penetration of pesticide residues on exposed dermal regions of face/forehead and neck at the end of work shift (21). Exposed forehead, face, and neck regions were wiped five times by repeatedly folding and turning wipe samplers by the trained personnel using surgical gloves on.

At the end of the sampling event, the samples of the patch, wipe, and hand washing were collected in the Ziploc bags closed by twisting the upper part of the bag to make an air-tight seal, labeled appropriately for each operator, and transported in chilled condition using ice-packs from the field to laboratory and stored at −20°C (deep-freezer HF 500 CHP; Carrier, USA) until extracted. All the extractions were completed not later than 7 days after the collection of samples.

### Assessment of dermal exposure

In the present study, measurements of dermal exposure were used to quantify the potential and actual dermal exposure of operators on each work shift. The potential dermal exposure (PDE) is defined as the total amount of pesticide in contact with the body surface of farmers, namely, protective clothing, work clothing, and uncovered skin; actual dermal exposure (ADE), in contrast, is the amount of pesticide in contact with the uncovered skin, and therefore, the fraction passed through protective and work clothing and that poses a risk of being percutaneous absorption (8, 21). All external and internal patches were used to estimate PDE and ADE, respectively, for the exposed body regions. The PDE and ADE were calculated using Equations (1) and (2), respectively. Further, to check the PDE calculations for the face and neck region, the skin wipes of the exposed region of the face and neck of the operator without using the face mask/PPE were considered, while for ADE calculations also the same procedure was followed, but after the use of face mask/PPE.


(1) $$\begin{array}{l} \text{PDE = Measured conc}.\;({\text{ng/cm}}^{2})\text{ on sampler attached}\\ \text{ over work clothes }\times \text{ Exposed anatomical area }({\text{cm}}^{2}) \end{array}$$

(2) $$\begin{array}{l} \text{ADE = Measured conc}.\;({\text{ng/cm}}^{2})\text{ on sampler attached over}\\ \text{ skin inside work clothes and PPE x Exposed anatomical}\\ \text{ area}\;({\text{cm}}^{2}) \end{array}$$

where measured ng/cm^2^ is the total value given for deposition and exposed dermal region for the patch or wipe sampler which makes the summation of surface area torso (7,100) [back (3,550) + chest (3,550)], arms (4,120) [upper arms (2910) + forearms (1,210)], legs (6,200) [upper legs (3,820) and lower legs (2,380)] and wipe (760) [face and forehead (650) and neck (110)], whereas the surface areas used include both right and left arms and legs of the adult body (80th percentile man) (8).

Further, the measured PDE was transformed to percentual PDE (%PDE) by normalizing the PDE value with the total amount of the active ingredient used, and expressed as a percentage, to allow comparisons between different trials, where different active ingredients and consequently dissimilar pesticide amounts were used (23). The %PDE was calculated using Equation (3):


(3) %PDE = [PDE / amount of active ingredient (mg)] x 100

The concentration of pesticide in each extract combined with the duration of each experience gives a time-rate value for the dermal exposure. The skin loading rate (μg h^−1^) was calculated from the operators' estimated number of hours of applications per day (24). From the questionnaire survey data, the estimated duration of the number of hours spent was also obtained.

For each operator, the percentage coverall penetration was calculated in terms of penetration factor (PF), which can be defined as the fraction of pesticides that cross the clothing barrier and is available for contact with the skin (25). Further, the resulting data of both PDE and ADE were used to calculate the percentage of PF using Equation (4):


(4) PF anatomical region (%) = [ADE / (ADE + PDE)] x 100

The margin of safety (MOS), a risk indicator, was measured as previously reported (26–28) for each tested pesticide residue using Equation (5):


(5) MOS = [AOEL × average body weight / (DE x AF)]

where DE is the total dermal exposure and AF is the absorption factor.

A value of MOS ≥1 would indicate safe working conditions, while the MOS <1, the unsafe conditions. If acceptable operator exposure level (AOEL) is not available, then no observed adverse effect level (NOAEL) was used based on the average body weight of 60 kg adult (Table 1). The AF value was taken as 0.11, which indicates the dermal absorption of 10%, with an addition of 1% extra to consider the inhaled fraction also, whereas DE is equal to the summation of PDE obtained from patch and wipe (μg) and final residues (μg) from washings of hands. Further, for MOS calculation, a “worst case scenario” was assumed by taking into account the practice of not using appropriate gloves and hence, any additional coefficient was not added to consider the use of protective measures (23). Therefore, the MOS was calculated using Equation (6).


(6) MOS = [AOEL x 60 / (DE x 0.11)]

**Table 1:** Key information about the pesticides used by operators.

**Operator**	**Trade name of pesticides**	**Amount of pesticides used (mg)**	**Active ingredient (%)**	**Chemical group**	WHO classification^*^	**AOEL^a^ or NOAEL^b^ (mg/kg bw/ day)^#^**
1V	Acemain/Acestar	250	Acephate (75% SP)	Organophosphate (OP)	II	0.0008^a^
2V	Orax	210	Profenofos (50% EC)	OP	II	1.0 ^b^
3V	Dhanulux	480	Quinalphos (25% EC)	Organothiophosphate	II	0.05^b^
1C	Hilban	220	Chlorpyrifos (20 % EC)	OP	II	0.001^a^
2C	Orax	300	Profenofos (50% EC)	OP	II	1.0^b^
3C	Monocil	180	Monocrotophos (36% SL)	OP	Ib	0.005 ^b^

* World Health Organization - classification of acute toxicity (2004): Ib-highly hazardous; II-moderately hazardous.

# Source: EU Database, 2012.

### Extraction procedure and instrumental analysis

The hand washing samples collected were filtered using Whatman filter paper (29) and then passed three times through anhydrous sodium sulfate. The filtrate was then completely evaporated to dryness using a rotary evaporator (AD 2C, Aditya Scientific, India) at 30°C and 80 rpm. The residues were reconstituted using 1 mL of acetonitrile. While the wipe and patch samples were also subjected to ultra-sonication (Ultrasonic Cleaner, Equitron, India) for 15 min using 20 mL of methanol. The methanol extract was transferred to a glass test tube and dried under a gentle stream of nitrogen using Turbo-Vap (LV concentrator, Caliper Life Sciences, India) at 30°C and 15 psi. Re-constitution was done using 1 mL of methanol. Both the extracts were then filtered into an auto-sampler vial using a 0.22 μm PTFE cellulose syringe filter (Nupore Filtration Systems, India), and stored at −80°C (ultra-low temperature freezer, Haier, China) until analyzed.

A liquid chromatography system (Shimadzu LC 20AD) equipped with a mass spectrometer (Applied Biosystems MDS Sciex 4000-Q TRAP triple quadruple) and auto-sampler (SIL-HTC model) controlled using Analyst Software (version 4.1.2) was used for the quantitative analyses and qualitative confirmation. The chromatographic separation was carried out on the Zorbax SB-C18 HPLC column (internal diameter of 4.6, 250 mm length, and 5 μm particle size), maintaining a minimum of 25°C and maximum of 85°C oven temperature. The analysis was done in the multiple reaction monitoring (MRM) positive turbo ion spray (ESI) mode with high resolution. Two mobile phases (mobile phase A – Milli-Q water containing 0.1% formic acid and mobile phase B - methanol with 0.1% formic acid) were used in gradient mode. Initially, Pump B was maintained at 10% for 0.01 min subsequently for 20 min, changed to 98% at 25 min, and again to 10% at 32 min giving a total run time of 32 min. A constant flow rate of 800 μL min^−1^ was maintained with an injection volume of 35 μL. The ion spray voltage (IS) of 5,500 eV was used and the interface heater was held at a temperature of 500°C.

### Quality control

The standardized method used for the quantitative and qualitative determination of OP in hand washings and patch/wipe samples was modified and the same was validated in-house prior to commencing the sample analyses (30); ICH Q2 (R1) guidelines (1995). Individual analyte standard was prepared by dissolving 1 mg of neat standard in 1 mL of acetonitrile:distilled water in a 1:1 ratio (1,000 mg L^−1^) and a working standard mixture of 20 mg L^−1^ was prepared from the stock solutions. Primary and secondary working solutions were prepared and TPP was used as the internal standard at 200 ng mL^−1^ concentration. All the standard solutions were sealed and stored at −80°C for future analyses. Mass parameters for OPs were optimized in multiple reaction monitoring (MRM) mode (Table 2). The absence of an analyte peak in the blank run indicates the selectivity of the method. The analytes showed consistent retention over 10 runs with a retention time variation of ±0.2 min and the RSD of the obtained peak areas over the 10 runs was observed to be <3%. The calibration plots obtained by plotting the peak area vs. analyte concentration for all the pesticides showed good linearity with correlation coefficients (r) ranging from 0.9986 to 0.9999. Performance parameters of the LC-MS/MS method for the determination of pesticide residues in hand washings (Table 3) and wipe/patch were determined (Table 4). Briefly, the concentration range used for hand washing varied from 0.5 to 1,000 ng mL^−1^, while that for wipe/patch was in the range from 0.2 to 1000 ng mL^−1^. The sensitivity of the method was evaluated by determining the experimental limit of detection (LOD) and the limit of quantitation (LOQ) for each analyte at a signal-to-noise ratio (S/N) of 3:1 and 10:1, respectively. It was found that the LOD ranges from 0.5 to 1 ng mL^−1^ in hand washing and 0.2 to 0.5 ng mL^−1^ in wipe/patch. The LOQ in hand washing and wipe/patch ranges from 1 to 5 ng mL^−1^ and 0.5 to 5 ng mL^−1^, respectively. The recoveries determined at two different concentrations were in the range from 75 to 102% for hand washing and from 95 to 107% for wipe/patch, which proves the accuracy of the method. The precision was determined as relative standard deviation (RSD) in terms of repeatability (intra-day) and reproducibility (inter-day) at three fortification levels (1, 50, and 500 ng mL^−1^) for hand washing and wipe/patch were ≤ 15%.

**Table 2:** Optimized MS/MS parameters for organophosphorus compounds in multiple reaction monitoring (MRM) mode using different energy profiles.

**Analyte**	**MRM transition (parent/quantifier)**	**DP**	**EP**	**CE**	**CXP**	**R_T_ (min)**
Acephate	184/143	46	10	11	12	8.0
Monocrotophos	224.1/127.1	46	6	21	12	11.9
Quinalphos	299.1/147	60	5	30	7	13.2
Profenofos	375/305	61	10	27	26	14.6
Chlorpyriphos	350/198	56	10	19	8	19.0
TPP (IS)	327.1/77.1	96	8	63	4	22.1

DP, De-clustering potential; EP, entrance potential; CE, collision energy; CXP, collision cell exit potential; R_T_, retention time.

**Table 3:** Performance parameters of the LC-MS/MS method for the determination of pesticide residues in hand washings.

**Analyte**	**LOD (ng mL^−1^)**	**LOQ (ng mL^−1^)**	**Precision at different concentration levels (%RSD)**						**% Recovery** ±**SD (*****n*** = **6)**	
			**Intra-day Rp**			**Inter-day Rc**				
			**1 ng mL^−1^**	**50 ng mL^−1^**	**500 ng mL^−1^**	**1 ng mL^−1^**	**50 ng mL^−1^**	**500 ng mL^−1^**	**50 ng mL^−1^**	**500 ng mL^−1^**
Acephate	0.5	5	2.1	3.8	3.2	4.3	10.7	3.8	96 ± 1	100 ± 2
Monocrotophos	1	2	3.3	3.5	5	14.7	6	2	94 ± 2	99 ± 3
Quinalphos	0.5	1	6.1	2.7	4.3	11.7	8.5	8.4	96 ± 1	97 ± 2
Profenofos	0.5	1	7.3	6.7	2.8	8.4	3.7	6.2	96 ± 3	98 ± 1
Chlorpyriphos	1	2	4.6	3.7	7.4	10.2	7.2	6.8	95 ± 3	79 ± 4

Rp, repeatability (n = 6); Rc, reproducibility (n = 6); SD, standard deviation; RSD, relative standard deviation.

**Table 4:** Performance parameters of the LC-MS/MS method for the determination of pesticide residues in wipe/patch.

**Analyte**	**LOD (ng mL^−1^)**	**LOQ (ng mL^−1^)**	**Precision at different concentration levels (%RSD)**						**% Recovery** ±**SD (*****n*** = **6)**	
			**Intra-day Rp**			**Inter-day Rc**				
			**1 ng mL^−1^**	**50 ng mL^−1^**	**500 ng mL^−1^**	**1 ng mL^−1^**	**50 ng mL^−1^**	**500 ng mL^−1^**	**50 ng mL^−1^**	**500 ng mL^−1^**
Acephate	0.2	0.5	2.7	3.0	1.8	13.3	3.9	4.7	104 ± 3	104 ± 2
Monocrotophos	0.5	5	7.6	3.3	2.9	7.2	12.6	10.9	104 ± 2	100 ± 3
Quinalphos	0.2	0.5	1.6	3.9	4.3	5.6	11.1	9.9	100 ± 3	101 ± 3
Profenofos	0.5	5	4.6	2.3	3.3	10.2	11.5	9.8	102 ± 2	100 ± 2
Chlorpyriphos	0.5	1	1.6	3.6	1.3	7.7	10.9	14.0	99 ± 3	98 ± 3

Rp, repeatability (n = 6); Rc, reproducibility (n = 6); SD, standard deviation; RSD, relative standard deviation.

### Statistical analysis

The raw data collected using the questionnaires and LC-MS/MS were coded, entered into specially designed databases (Microsoft Access), and transferred to appropriate spreadsheets (Microsoft Excel) for statistical analysis using the SPSS software (version 23). The descriptive variables were represented as mean (standard deviation), frequency, and percentages. A statistical correlation was determined among different exposed dermal regions of the operators, before and after the use of PPE. Therefore, the t-test was carried out to assess the association between exposure levels among the operators before and after the use of PPE for different exposed dermal regions, and the associations were studied with a 95% confidence interval (CI) and, statistical significance was considered at *p* < 0.05.

## Results

### Field observations

All the operators were men and mean age was 35.2 years with an average farming experience of 16.2 years. Further, they were marginal farmers with a land holding of 2.66 acres.

The field observations and pesticide management data collected for the present study involved a single event of pesticide treatment for each operator on each separate occasion. The operators were asked to carry out the pesticide spraying operations as how they practiced and as always. It was observed that the five OPs were the most commonly applied insecticides which were registered under the Insecticides Act for use in the country (31), using hand-pressurized knapsack spraying devices (backpack pump with hand or motorized spray) which were carried on their back. It was found that the sprayings were done with the lance positioned in front of the operators, while they walk forward in different directions in the treated field areas. Further, the spraying activities were found to have been done in the morning time between 7 and 10 a.m., when the temperature was relatively cool. It was found that the number of pesticides sprayed was not done as per the Good Agricultural Practices (GAPs) and also varied as per the crop cultivated, the intensity of the pest infestation and the area to be treated. Also, none of the six operators used any PPE of their own while handling the pesticides, except one who was found to have covered his head/mouth using a handkerchief. The discomfort in using the PPE coupled with un-affordability and inaccessibility was found to be some of the self-reported major reasons for not using the PPE. Further, no one was found to have taken training from authorized agricultural officials. Also, all the operators stored pesticides at farms in a separate shed. Further, they were also disposing of empty containers after their use without even rinsing the same in the agricultural fields in which they were performing the agricultural activities. Details of meteorological conditions were recorded indicating high temperature and low humidity throughout the duration of the operators' work in the field (Table 5). It was also observed that they sprayed pesticides against the wind direction.

**Table 5:** Details of operational modalities for each pesticide treatment at the field level.

**Operator**	**Active ingredient**	**Crop under cultivation**	**Work period (min)**	**T (°C)^a^**	**RH (%)^a^**	**Wind speed (km/h)^a^**	**Garments**	**Potential regions of body that can get exposed**
1V	Acephate	Tomato	20	32.3	51.4	7.4	Long trousers, long sleeved cotton shirt, rubber shoe	Head, face, neck, hands
2V	Profenophos	Eggplant	25	34.3	50.8	9.4	Short trousers, long sleeved cotton shirt	Head, face, neck, hands, feet
3V	Quinalphos	Okra	80	35.6	46.4	8.7	Long trousers, long sleeved cotton shirt	Head, face, neck, hands, feet
1C	Chlorpyrifos	Cotton	30	33.4	42.2	6.6	Long trousers, short sleeved T-shirt, casual shoe	Head, face, neck, hands, forearms
2C	Profenophos	Cotton	20	30.9	47.6	9.3	Long trousers, short sleeved shirt	Head, face, neck, hands, forearms, feet
3C	Monocrotophos	Cotton	37	36.4	52.5	7.9	Short sleeved shirt, sarong	Head, face, neck, hands, forearms, lower legs, feet

a Mean of work period.

### Pesticide management

The pesticide management practices were undertaken in three phases: the preparation of the pesticide followed by the application, and the cleaning of the spraying equipment (Table 1). The operator took about 15 to 60 min for completing the farming tasks of mixing, loading, spraying, cleaning the sprayer, removing work garments, etc. However, it depends on the area of the field and the quantity of pesticides to be applied.

The preparation involves mixing up the pesticide formulation with water, followed by loading it into the tank of the knapsack sprayer. While in the case of solid formulation, they were mixed with their bare hands in approximately 10 to 15 L of water in a tank of 20 to 50 L capacity without following any GAPs. Of the six operators chosen for the study, only one was found to have mixed the solution with the aid of a wooden stick, while the rest mixed with bare hands. The mean concentration of the active ingredient in the liquid mixture was found to be 21.87 mg/L. It was observed that there were some technical errors during the preparation of the pesticide formulation such as spillages, overflow of tanks with excessive foaming, blockage of pipes, etc. due to which the formulations were found to be directly coming in contact with the operator's body such as hands, arms, chest, and legs.

The pesticide application starts with the knapsack sprayer being mounted on the back of the operator to initiate the spraying in the field. During this process, it was found that the operators' body was exposed to the droplets emitted by the nozzles of the knapsack sprayer if the operator was using a defective sprayer. The hand pressure sprayer used for spraying was found to be 10 years old and was hardly rectified for its leakages. Operators were found to be spraying with the lance approximately 30 cm above the top of the crop in front of them by swinging it from side to side, which will, in turn, form the spray aerosol in front of him into which he walks forward. The spray tank usually has a high-discharge nozzle that discharges pesticide formulation at a pressure of 0.90 ± 0.18 L min^−1^. It was further found that the pesticides applied to the crop ranged from 180 to 480 mg acre^−1^. It was further observed that most of the operators (67%), before initiating the spraying task/entering the agricultural field, were checking the speed of the spray nozzle to close proximity and keeping the spray machine in “on mode” resulting in the splashes of pesticide solutions falling onto their body parts like chest, face, hands, and eyes. Further, to clear blockages of the nozzle if any, they were found to be blowing the air through their mouths which will have a direct impact on the operator's health due to unsafe handling practices/GAPs (Figure 2).

Cleaning was done by pouring the clean water from the nearby water tank/tap to ensure that all the accessories of the tank were washed thoroughly and it was repeated at least two times. During this process, it was observed that the spillage from the washings of the equipment fell on the operator's body, as they do not wear any protective gear.

Further, most of the operators were found to have re-entered the treated/sprayed fields within 2 days of application without following any proper protection. Apart from working on their own land, the operators were found to have been engaged in the farming activities such as spraying, planting, pruning, weeding, threshing, cutting, picking, and harvesting on other farms also. They were also found to be using the same clothes used during spraying till the next spray without washing them and this may result in possible substantial exposure to the pesticides. On the whole, it was noted that on average, the operator was spending 6.2 hours per day on farming activities.

### Pesticide residues concentration in hand washing

The hand washings evaluated for each operator were found to be the major contributors compared to the overall dermal exposure. From the results, it could be revealed that the residue levels ranged from 0.07μg (operator 2C) to 53.2μg (operator 2 V) among those who worked without using gloves, while, a reduction in the residues was found among those who used gloves (0 to 1.12μg) (Table 6).

**Table 6:** Pesticide residues levels in hand washings (μg) among operators before and after use of PPE.

**Operator**	**Before use of PPE**	**After use of PPE**
1V	1.65	0.952
2V	44.8	0.1064
3V	53.2	0.264
1C	0.206	0
2C	0.07	0
3C	2.16	1.122

### Assessment of dermal exposure

#### Potential and actual dermal exposure

The data on dermal exposure, representing the results of PDE, ADE, percentual dermal exposure, and loading rates are summarized (Table 7). Of the different exposed dermal parts of the body, the PDE and %PDE levels were found to be more in the torso parts of the operator followed by arm, face, and neck regions. Overall, the PDE values ranged from 0.15 to 13.45 μg, while in contrast a reduction was found in exposure levels in ADE as compared to PDE (0 to 0.629 μg). The zero value here suggests the partial protection provided by the PPE against pesticide exposure. After considering the duration of exposure to be 6.2 h per day, the skin loading rates, PDEh and ADEh, ranged from 0.024 to 2.17 μg/h and 0 to 0.026 μg/h respectively. It was further found that the dermal exposure values were also influenced by the type of the crop that was cultivated, as the mean (SD) values were found to be 4.19 (0.19) among the vegetable cultivators, while it was 1.12 (0.4) among the cotton cultivators.

**Table 7:** Potential and actual dermal exposure (PDE and ADE, expressed in μg), %PDE, %ADE and skin loading rates for all operators.

**Operator**	**Exposed dermal regions**	**PDE (μg)**	**%PDE**	**PDEh (μg/h)**	**ADE (μg)**	**%ADE**	**ADEh (μg/h)**
1V	Arm	5.614	2.25	0.905	0.629	0.25	0.102
	Leg	0.265	0.11	0.043	0.022	0.01	0.003
	Torso	2.9	1.16	0.468	0.16	0.06	0.026
	Face + neck	13.452	5.38	2.17	0.138	0.06	0.022
2V	Arm	3.368	1.6	0.543	0	0	0
	Leg	0.837	0.4	0.135	0	0	0
	Torso	9.124	4.34	1.472	0	0	0
	Face + neck	0.337	0.16	0.054	0.056	0.03	0.009
3V	Arm	6.716	1.4	1.083	0	0	0
	Leg	0.901	0.19	0.145	0	0	0
	Torso	6.284	1.31	1.013	0	0	0
	Face + neck	0.477	0.1	0.077	0.16	0.03	0.026
1C	Arm	0.49	0.22	0.079	0.002	0	0
	Leg	0.149	0.07	0.024	0.007	0	0.001
	Torso	1.111	0.51	0.179	0.005	0	0.001
	Face + neck	0.92	0.42	0.147	0	0	0
2C	Arm	0.561	0.19	0.091	0.006	0	0.001
	Leg	0.147	0.05	0.024	0.023	0.01	0.004
	Torso	0.536	0.18	0.086	0.035	0.01	0.006
	Face + neck	4.051	1.35	0.653	0	0	0
3C	Arm	0.558	0.31	0.09	0.026	0.01	0.004
	Leg	0.815	0.45	0.132	0.047	0.03	0.008
	Torso	2.574	1.43	0.415	0.123	0.07	0.02
	Face + neck	1.581	0.88	0.255	0	0	0

**Figure 1:**
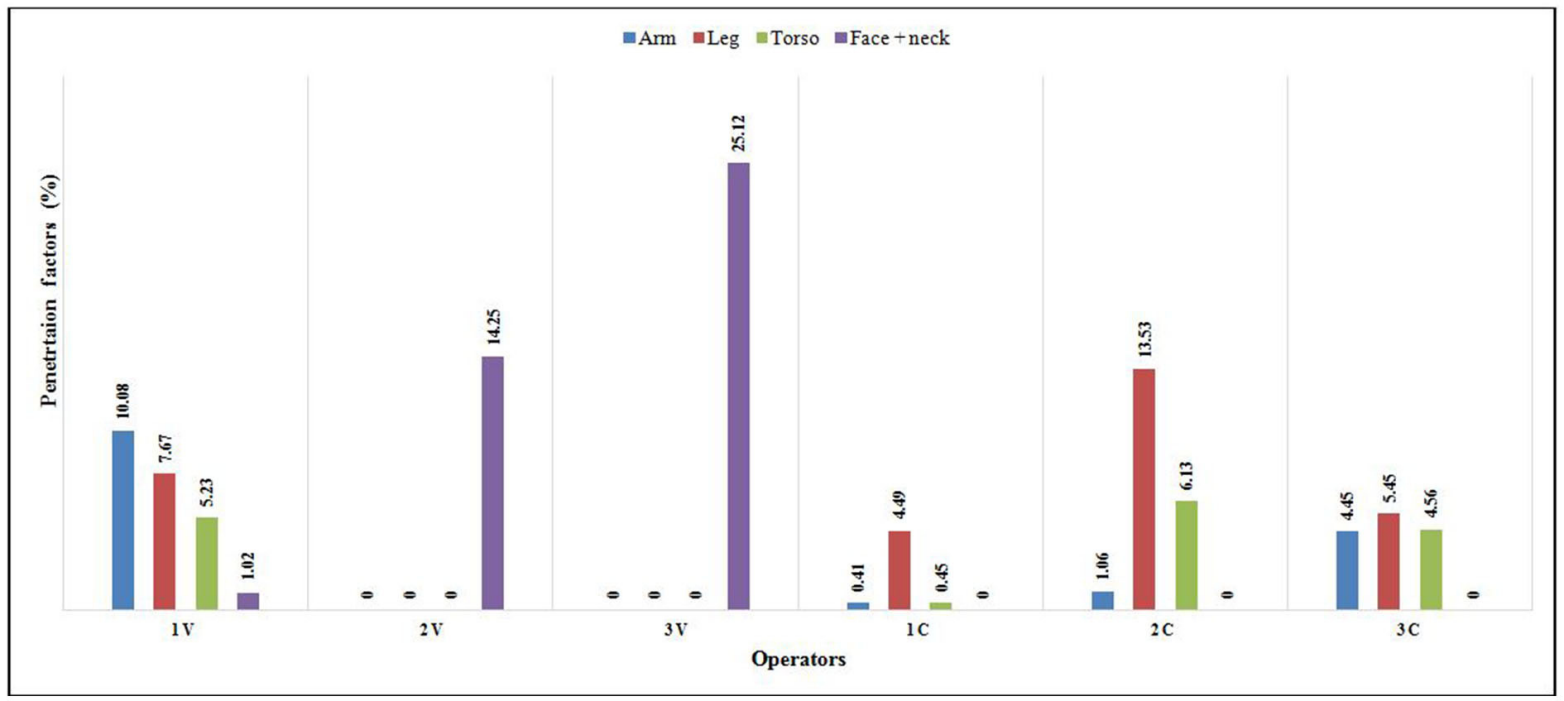
Penetration factor for each anatomical region.

PF values of operators who worked with a complete set of PPE (Tyvek coverall, full face mask, boots, and gloves) ranged from 0.0 to 25.1%. Negligible values of PF of arm, leg, and trunk for the operators 2V and 3V and for face and neck for the operators 1C, 2C, and 3C indicate complete body protection from dermal exposure to pesticides; in these cases, PPE functioned as a complete barrier to the penetration of pesticides and provides absolute protection. For other operators, the protection was not complete as the mean values of PF ranged from 2.67% for arm to 6.73% for the face and neck region (Figure 1).

**Figure 2:**
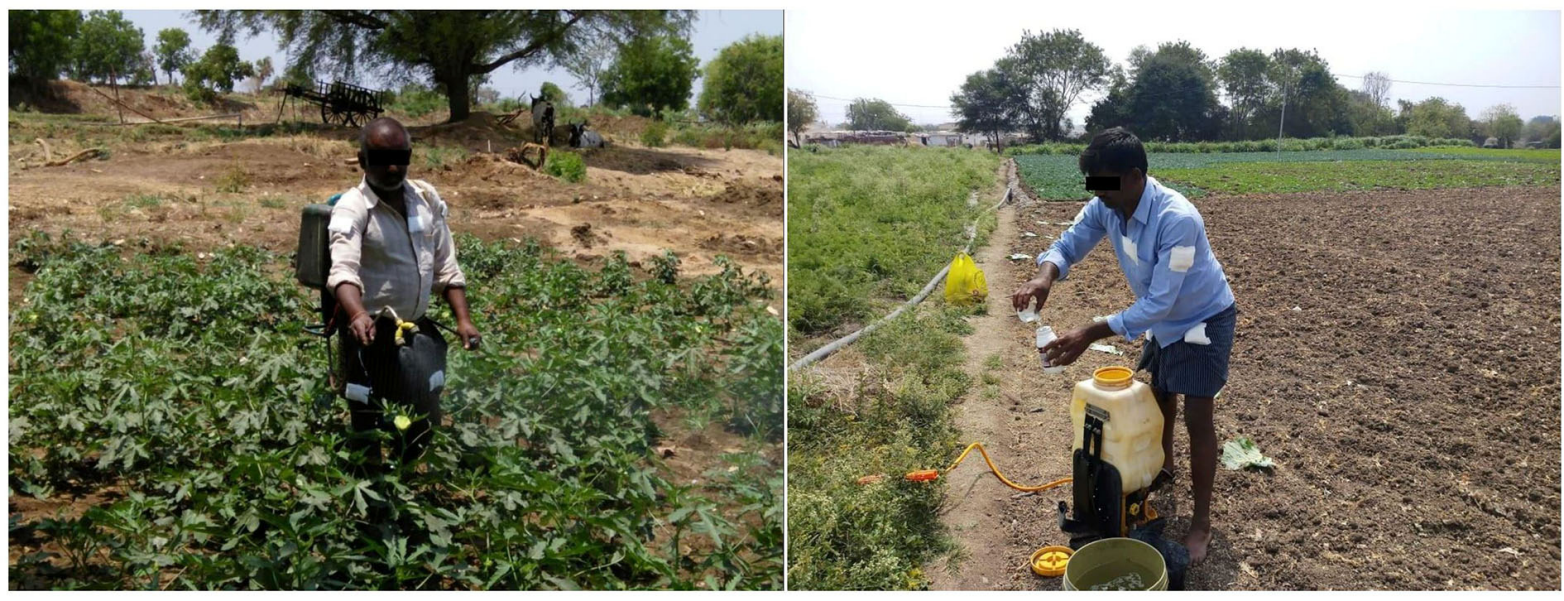
Operators mixing and spraying pesticides without following any safety protocol.

MOS was calculated for each case to determine if the spraying operation was done by following safe handling practices or not. It was observed that out of the six operators studied, four had not adopted safe handling practices (Table 8).

**Table 8:** MOS for different pesticides.

**Operator**	**Crop**	**Pesticide used**	**MOS**
1V	Tomato	Acephate	0.02
2V	Eggplant	Profenophos	9.33
3V	Okra	Quinalphos	0.40
1C	Cotton	Chlorpyrifos	0.19
2C	Cotton	Profenophos	101.67
3C	Cotton	Monocrotophos	0.35

**Figure 3:**
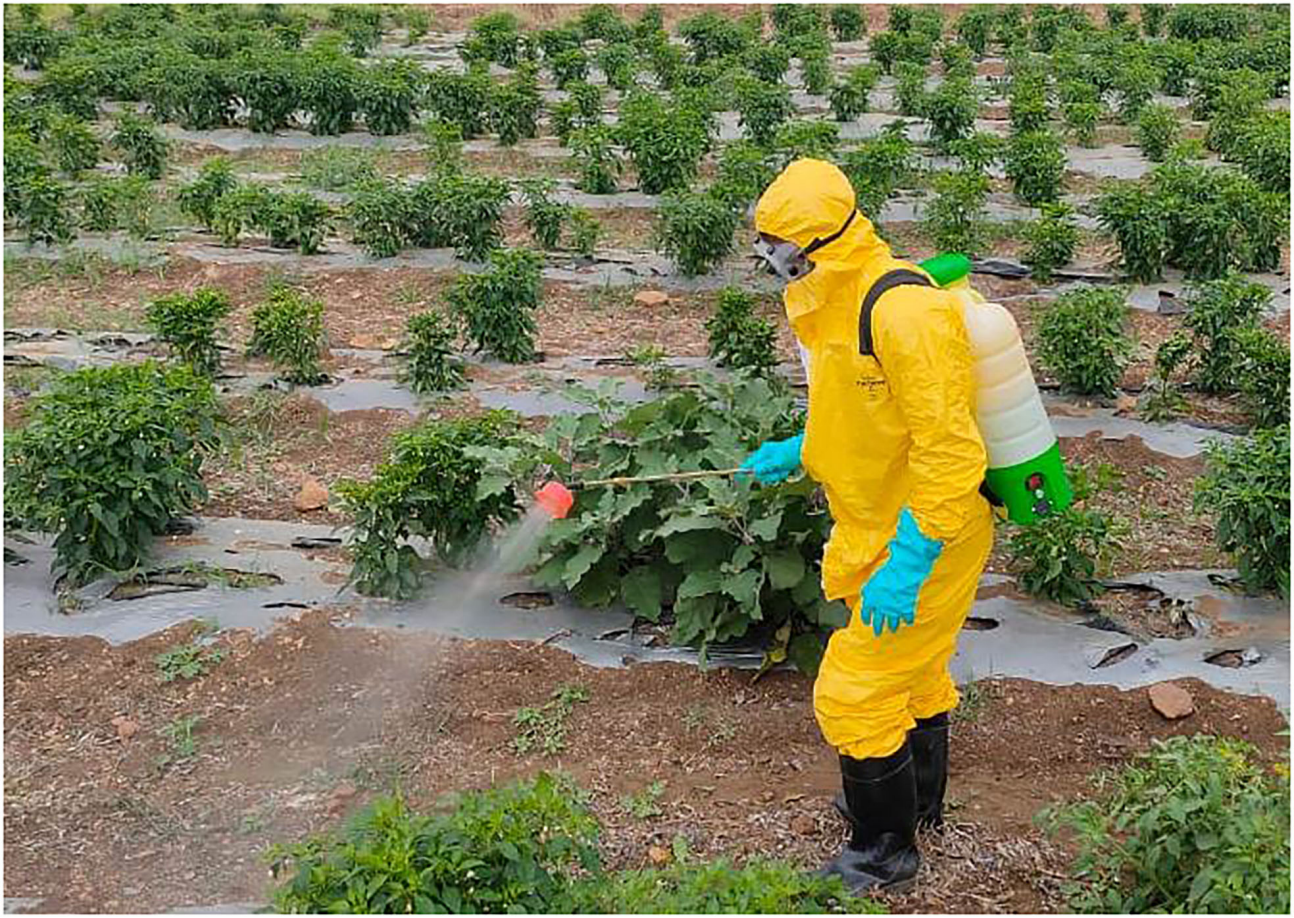
Operator involved in spraying activity after wearing provided PPE.

Results revealed a significant difference (*p* < 0.05) concerning exposure levels in leg and torso regions among operators who have used PPE (Table 9 and Figure 3).

**Table 9:** Association among different exposed dermal regions of the operators.

**Exposed dermal regions**	**Before use of PPE**		**After use of PPE**		***t-*value**	***p-*value**
	**Mean (μg)**	**SD**	**Mean (μg)**	**SD**		
Face + neck	3.470	5.076	0.059	0.073	1.646	0.161
Arm	2.885	2.790	0.111	0.254	2.426	0.059
Hand washing	17.014	24.931	0.407	0.500	1.631	0.164
Leg	0.519	0.367	0.017	0.018	3.347	0.020^*^
Torso	3.755	3.308	0.054	0.070	2.740	0.041^*^

* Statistical significance at p < 0.05 and CI at 95%.

## Discussion

In the present study, the dermal exposure was accessed among the vegetable and cotton crop cultivators using and not using PPE while engaged in farming activities in Rangareddy district, Telangana, Southern India. From this study, it was evident that the dermal contamination was found to be relatively less among those who have used PPE. For the last six decades, researchers have stressed that the adoption of GAPs and the use of PPE are the ideal methods to minimize the risk of exposure (32, 33).

Assessment of an operator's dermal exposure to pesticide is a very critical task as it depends on multiple factors such as type of equipment used, type and quantity of pesticide formulation used, their application rate, duration of application, climatic conditions prevailing at the time of application, use of PPE, their attitude in following safety measures, and any training undertaken as per the GAPs (33). In the present study, self-reported information gathered on the operational modalities of pesticide handling both in terms of field observations and the pesticide management practices adopted by the operators provided possible evidence of likely substantial exposures at all phases of pesticide handling during spraying and other agricultural activities. In the Indian agro-economy, the majority (85%) are small and marginal farmers, who have no access and cannot afford to use the automated spraying equipment is lacking as part of GAPs and is being observed in the present study also (34).

Further, the operators were also observed to undertake spraying activities with bare bodies sometimes to avoid the heat in the prevailing tropical climatic conditions, which would have increased the rate of dermal penetration. In the present investigation, the unsafe agricultural practices such as blowing the nozzle of the knapsack sprayer with the mouth, re-entry into the treated fields/crops at short intervals, and consuming the food/drinking water near the sprayed field areas followed by the lack of PPE use by the operator might aid in exacerbating the exposure resulting in the elevated PDE values. This may be attributed to poor training and handling practices, technical knowledge on the safe use of pesticides, lack of awareness of the hygienic practices, and inadequate knowledge on the adoption of protective measures during spraying coupled with low education levels. Similar observations were reported by earlier researchers among the pesticide handlers (35). Further, it was also observed from the present investigations that the operators were not considering the meteorological parameters such as the direction of the wind, humidity, temperature, etc., recorded on the day of spraying, which also would influence the drifting of the pesticide residual droplets followed by volatility which will not only affect the environment but also the perspiration rate of the operators (7).

Widespread use of the dosimeter and hand washing methods can be observed in earlier studies for assessing dermal exposure, since these methods have the clear advantage of low capital costs and ease of use (35–37). From the present study findings, it was observed that contamination through hands was found to have been the major contributor to all the dermal exposure parameters that were analyzed among the operators, who have not followed any GAPs. Further, in the present study, it was observed that all the operators have used liquid formulations of pesticides for spraying purposes. It was further found that exposure through hands accounted for >62% of the total dermal exposure, which would have been due to the operators mixing/loading with bare hands when using a liquid formulation of pesticides for spraying purposes, touching the spraying equipment frequently, and/or most of the times due to the deposition of droplets on hands from spray clouds/drifts. Studies conducted earlier reported that the exposure via hands often accounted for a significant portion of total dermal exposure (35, 38). A study reported that the exposure through hands was found to be 22–62 times greater than that of the solid formulation of pesticides when mixed and loaded with the liquid formulation of pesticides (23). From the current study, it was found that hand contamination was the highest contributor among the operators who have not used gloves and found to be in line with the previous findings reported among those who have not used PPE, there was found only slight contamination through hands among those operators who have used gloves (26).

In the present investigation, the PDE levels evaluated in the patch and wipe methods revealed that the torso region (14%) followed by the arm and the face/neck regions (13%) were the major contributors to dermal exposure among most of the operators. A significant reduction of the pesticide residual concentration was found in leg and torso regions among the operators who have used the PPE for 90 days. It was further found that facial exposure was another important dermal region for exposure among most of the operators as they were found to be frequently wiping their sweat on their faces with their contaminated bare hands. Of the different kinds of exposure, the exposure of the head and face was found to have been rarely reported as an important component of pesticide exposure, although this route was identified as one of the major contributors to dermal exposure among the hand-held applicators (39). Further, higher levels of percentual dermal exposure (% PDE) were found among the operators, while showing a reduction in the same after using the PPE (%ADE). Further, it is noteworthy that the lower values of ADE, %ADE, and ADEh indicate the importance of using PPE, which has also been reported by earlier researchers (40, 41).

Further, it was also found from the present study that the PDE values were higher among the vegetable cultivators as compared to the cotton cultivators as different crop heights and densities can explain the differences in the mean PDE values for different crop cultivators. Though the influence of different crops on the exposure amount and distribution pattern has been previously investigated elsewhere, the crop-wise distribution of PDE values reported among the Indian farmers is meager (42, 43). In the present investigation, the PF values vary among the operators, probably due to differences in pesticide handling methods and the types of different classes/groups of the pesticides used, and the type of work clothing that was used which might have determined the penetration and thereby having an impact on the exposure (25, 44). Further, in the present study, the higher PF values for the face, neck, and lower parts of the body (upper and lower legs) were found to have agreed with the earlier finding (36). This indicates that the PF depends not only on the actual use of the PPE but also on the proper use of PPE as the penetration of the pesticide residues among the operators in the present study has been observed to be more, if the closure of the coverall is incomplete or wearing cloth with sleeves rolled-on while during spraying and/or frequent opening/closing of the masks in between during the spraying operations and thereby paving the way for the penetration of pesticide residues through seams and zips (45).

Of the various indicators that were used to assess the dermal exposure among the operators in the present study, the MOS was found to be a better indicator than the PDE, as the risk estimation is strongly affected by the toxicological properties such as AOEL/NOAEL for each active ingredient, as the exposure levels cannot be considered as safe or unsafe based on the PDE values. Further, the MOS establishes a comparative frame under different field situations such as the types of different pesticides used/concentrations applied/application techniques adopted, etc. (43). Results from the present study revealed that 67% of the operators were found to have adopted unsafe practices, emphasizing the associated risk. The limitation of the study is that it has been done using a smaller sample size; however, a large prospective study is warranted to validate the findings of the present field trial with a larger sample size and also to assess the exposure impact on gender. Additionally, the persistence of the pesticide residues in the body fluids among the exposed is also needed to be undertaken in order to assess the adverse health effects.

## Conclusion

With the use of the patch dosimetry, hand washing, and wipe technique, the present field trial study highlights the dermal exposure to pesticides among Indian farmers in a real-time field scenario. The data on field observation and pesticide management indicate the variability in operative modalities among the operators and majority of them demonstrated an insufficient level of risk perception. Study results revealed higher PDE, %PDE, and PDEh levels and unsafe working conditions, as reflected by the low MOS risk evaluation which demonstrates that it is reasonable to expect possible health effects for farmers engaged in farming activities regularly without wearing PPE and by not adopting any specified GAPs. Further, the evaluation of dermal exposure after the use of supplied PPE by the operators in the trials indicated lower ADE, %ADE, and ADEh levels, highlighting the use of adequate PPE as a major important parameter for the operators' safety. To the best of our knowledge, so far the assessment of dermal exposure among Indian farmers using dosimeter and hand washing methods was not studied as part of the dermal exposure assessment. The exposure dataset from the present study could be used as a surrogate for the estimation of the operator's dermal pesticide exposure under similar pesticide use scenarios. This might also help in developing databases for risk assessment through dermal penetration/absorption and emphasizing the need for thorough training and comprehensive understanding of the safe handling practices to protect them from exposure.

## Data availability statement

The raw data supporting the conclusions of this article will be made available by the authors, without undue reservation.

## Ethics statement

The studies involving human participants were reviewed and approved by the Ethical Committee of the Indian Council of Medical Research - National Institute of Nutrition, Hyderabad, India (REF NIN Protocol number 11/I/2016). The participants/farmers/operators provided their written informed consent to participate in this study.

## Author contributions

SL participated in the analysis of the data and interpretation of the results and wrote the first draft of the manuscript. PJ contributed to the conception and design of the study. PJ, SM, and BJ wrote sections of the manuscript. JV, PY, AP, and MN were involved in the fieldwork, sample collection, and processing. All authors contributed to manuscript revision, read, and approved the submitted version.

## Funding

This work was funded by the Department of Science and Technology (DST)—Science for Equity, Empowerment, and Development (SEED) Division, Ministry of Science and Technology, Government of India (SEED/WS/004/2015).

## Conflict of interest

The authors declare that the research was conducted in the absence of any commercial or financial relationships that could be construed as a potential conflict of interest.

## Publisher's note

All claims expressed in this article are solely those of the authors and do not necessarily represent those of their affiliated organizations, or those of the publisher, the editors and the reviewers. Any product that may be evaluated in this article, or claim that may be made by its manufacturer, is not guaranteed or endorsed by the publisher.
